# Extraction, Rheological, and Physicochemical Properties of Water-Soluble Polysaccharides with Antioxidant Capacity from *Penthorum chinense* Pursh

**DOI:** 10.3390/foods12122335

**Published:** 2023-06-10

**Authors:** Yi Chen, Li Song, Pei Chen, Huiping Liu, Xiaowei Zhang

**Affiliations:** State Key Laboratory of Food Nutrition and Safety, Ministry of Education of China, College of Food Science and Engineering, Tianjin University of Science and Technology, Tianjin 300457, China; 16622881022@163.com (Y.C.); chenpei689689@163.com (P.C.); zhangxw@tust.edu.cn (X.Z.)

**Keywords:** *Penthorum chinense* Pursh polysaccharides, extraction, rheological properties, physicochemical properties, antioxidant activity

## Abstract

This study aimed to isolate polysaccharides from *Penthorum chinense* Pursh and evaluate their rheological characteristics, physicochemical properties, and antioxidant activity. The optimal conditions for the maximal extraction yield of *Penthorum chinense* Pursh polysaccharides (4.05 ± 0.12%) were determined by employing a single-factor test and response surface methodology which included an extraction time of 3 h, a liquid–solid ratio of 20 mL/g, and three separate extraction times. The rheological experiments showcased that the *P. chinense* polysaccharides exhibited typical shear-thinning behavior, with their apparent viscosity being influenced by various parameters such as concentration, pH, temperature, salt content, and freeze–thaw. The purified polysaccharides (PCP-100), having an average molecular weight of 1.46 × 10^6^ Da, mainly consisted of glucose (18.99%), arabinose (22.87%), galactose (26.72%), and galacturonic acid (21.89%). Furthermore, the PCP-100 exhibited high thermal stability and displayed an irregular sheet-like morphology. Its superior reducing power and free radical scavenging ability implied its significant antioxidant activity in vitro. Collectively, these findings provide important insights for the future application of *P. chinense* polysaccharides in the food industry.

## 1. Introduction

The extracts of natural plants widely distributed in nature, such as flavonoids, saponins, and polysaccharides, exert significant influences on many diseases’ pathological processes, as well as the aging process [1,2]. Among them, polysaccharides, as natural polymers composed of ketoses or aldoses linked by glycosidic bonds, have been proven to exhibit varied biological activities including hypoglycemic, antioxidant, antitumor, and anticoagulating activities [3,4]. Previous studies have indicated that the extraction methods are closely associated with the yields, biological activities, and physicochemical properties of polysaccharides [5]. Among these methods, hot water extraction technology has been widely chosen for the extraction of polysaccharides owing to its simple operation, low price, and easy mass production [6]. The exploration of rheological properties, which play multiple crucial roles in processes such as drying, blending, calefaction, and filtration, is beneficial for the application of natural polysaccharides in the food and pharmaceutical industries [7]. It is well accepted that the rheological properties of polysaccharides vary with various variables, such as shear rate, concentration, temperature, pH, and ionic strength, and so it is highly important to clarify the effects of single or combined factors [8].

For centuries, traditional herbal plants have been acknowledged as bountiful sources of polysaccharides. At present, several polysaccharides have been isolated and identified from various parts of herbal plants, such as branches, seeds, leaves, roots, fruits, and flowers [9]. *Penthorum chinense* Pursh, named “Ganhuangcao” in Chinese, is an edible plant belonging to the Saxifragaceae family and is predominantly found in Eastern Asia [10]. For millennia, the Hmong people settled in Gulin County, Luzhou City, Sichuan Province, have referred to *P. chinense* as the “immortal herb” [11]. Nowadays, the consumption of *P. chinense* tea has been increasingly adopted to alleviate symptoms caused by excessive alcohol intake and to treat conditions such as cholecystitis and infectious hepatitis. Several studies have reported the presence of diverse bioactive compounds in *P. chinense*, including flavonoids, steroids, polysaccharides, phenylpropanoids, and organic acids [12,13]. Recently, *P. chinense* polysaccharides have attracted growing scientific attention. Some scholars have successfully separated *P. chinense* polysaccharides via water extraction and enzymatic hydrolysis, respectively. Moreover, the structures and biological activities of *P. chinense* polysaccharides have been further explored [14,15]. Cold-water-soluble *P. chinense* polysaccharides were prepared in our previous study, and their anti-hepatocellular carcinogenic activity was also validated both in vitro and in vivo [16].

However, relatively little research has been performed on *P. chinense* polysaccharides compared to flavonoids and polyphenols. Interestingly, there are few reports about the physicochemical properties and rheological behaviors of *P. chinense* polysaccharides. Therefore, we optimized the hot water extraction process for *P. chinense* polysaccharides and further evaluated the effects of multiple parameters on their rheological behaviors. Subsequently, a homogeneous polysaccharide (PCP-100) was isolated by column chromatography and its physicochemical properties were analyzed based on its molecular weight, chemical composition, surface functional groups, and monosaccharide composition. The crystalline structure and surface morphology of PCP-100 were observed by X-ray diffraction (XRD) and scanning electron microscopy (SEM), respectively. In addition to its thermodynamic properties, the antioxidant activity of PCP-100 was further studied to assess the potential application of *P. chinense* polysaccharides in the food industry.

## 2. Materials and Methods

### 2.1. Materials

*P. chinense* leaves were obtained from Gulin, Luzhou, Sichuan, China. Bovine serum albumin (BSA), galacturonic acid, glucose, T-series dextran standards, standard monosaccharides, Sephadex G-200, ascorbic acid (V_C_), 2,2′-Azinobis-(3-ethylbenzthiazoline-6-sulphonate) (ABTS), and a total phenol assay kit were purchased from Solarbio Science & Technology Co., Ltd. (Beijing, China). Calcium chloride (CaCl_2_), sodium chloride (NaCl), salicylic acid, 2,2-Diphenyl-1-picrylhydrazyl (DPPH), potassium ferricyanide, ferric chloride (FeCl_3_), and ferrous sulfate (FeSO_4_) were supplied by Macklin Biotechnology Co., Ltd. (Shanghai, China). All other reagents utilized were of analytical grade.

### 2.2. Preparation of Polysaccharide

Before extraction, dried *P. chinense* leaves were smashed into a powder, sieved through a 60-mesh sieve, and then refluxed with petroleum ether to remove the lipids. The defatted powder was mixed with ultrapure water at different liquid–solid ratios of 5~40 mL/g, followed by extraction at 100 °C for different times (1~5 h). After repeated extractions for different times, the supernatant was collected by centrifugation and concentrated in vacuo by rotary evaporation. Subsequently, anhydrous ethanol was added to the concentrate until reaching a 60% final concentration, and the mixture was incubated at 4 °C to collect the polysaccharide precipitation. After being washed with ether, acetone, and anhydrous ethanol, the polysaccharides were thoroughly mixed with Sevage reagent (chloroform: n-butanol = 4:1) to remove the proteins. Next, the polysaccharide solution was transferred to a dialysis bag with a 10 kDa cut-off and dialyzed with ultrapure water for 72 h. The dialysate was collected and lyophilized to obtain the polysaccharides as HPCP. The yield of HPCP was calculated as follows: yield (%) = weight of HPCP (g)/weight of dry powder (g) × 100. Finally, the HPCP was further purified on a SephadexG-200 column (16 mm × 40 cm) and eluted with ultrapure water to obtain the purified polysaccharide named PCP-100.

### 2.3. Single-Factor Experiment Design

In this study, the effects of extraction time (1~5 h), liquid–solid ratio (5~40 mL/g), and number of extraction times (1~5) on polysaccharides yields were investigated. Only one parameter was changed in each experiment while holding the others constant. All experiments were conducted in triplicate.

### 2.4. Response Surface Experimental Design

According to the results of the single-factor analysis, the liquid–solid ratio, extraction time, and number of extraction times were used as the three variables (X_1_, X_2_, and X_3_) and grouped into three levels (−1, 0, and +1). The 17-run Box–Behnken experimental design (BBD) with three-factor and three-coded levels are shown in Table 1. Meanwhile, the relationships of the variables were assessed by quadratic model.

### 2.5. Rheological Determination

A rheometer (MARS 60, Haake, Vreden, Germany) equipped with heating system and a 35 mm 1° taper plate geometry (53 μm gap) was used to perform the steady shear viscosity tests. Various concentrations (0.5~10%, *w*/*v*) of the HPCP were dissolved in ultrapure water, after which the apparent viscosities were measured over a shear rate between 1 and 1000 s^−1^ at 25 °C. The effects of other parameters such as pH (1, 3, 5, 9, and 11), NaCl and CaCl_2_ concentrations (0.05%, 0.1%, 0.25%, 0.5%, and 1%), freeze–thaw treatment, and temperature (5 °C, 25 °C, and 45 °C) on the apparent viscosity of 4% HPCP were evaluated under the same conditions. In addition, the effect of temperature on the apparent viscosity of 4% HPCP was analyzed at a shear rate of 10 s^−1^ and a heating rate of approximately 10 °C/min.

### 2.6. Characterization Analysis of the PCP-100

#### 2.6.1. Chemical Composition Analysis

Taking glucose as the standard, the total carbohydrate content was assessed by the phenol-sulfuric acid method [17]. In addition, the content of uronic acid in the PCP-100 was measured using the m-hydroxydiphenyl colorimetric method with galacturonic acid as the standard [18], while that of the protein was determined according to the Bradford method by using bovine serum albumin as the standard [19]. The total phenolic content was measured with the Folin–Ciocalteu method using gallic acid (GAE) as a reference [4]. The ultraviolet and visible spectrum (UV) were recorded from 200 nm to 400 nm.

#### 2.6.2. Molecular Weight Analysis

High performance gel permeation chromatography (HPGPC) (Agilent Co., Santa Clara, CA, USA) was applied in the determination of the homogeneity and average molecular weight of the PCP-100, which was combined with a refractive index detector (RID) and a TSK-gel G4000 PWxl column (7.8 mm × 300 mm). After filtration through a 0.22 μm membrane, 20 μL of PCP-100 solution (0.8 mg/mL) was injected into the HPGPC system and carried out with ultrapure water at flow rate of 0.6 mL/min. The average molecular weight was calculated from a calibration curve, which was constructed by reference to T-series dextran standards (T10, T40, T70, T500, and T2000).

#### 2.6.3. Fourier Transform Infrared Spectroscopy (FT-IR) Analysis

The characteristic groups of the PCP-100 were captured by an FT-IR spectrometer (VECTOR-220, Bruker Co., Ettlingen, Germany) in the range of 4000 to 400 cm^−1^. Briefly, 150 mg of KBr was mixed with 1 mg of PCP-100 and then pressed into a KBr-polysaccharide pellet for measurement.

#### 2.6.4. Monosaccharide Composition Analysis

Ion chromatography was employed to determine the monosaccharide composition of the PCP-100, and it was coupled to a Dionex Carbopac PA20 column (150 mm × 3 mm) and a Dionex pulsed amperometric detector. The PCP-100 (1 mg) was hydrolyzed by adding trifluoroacetic acid (2 M, 1 mL) for 3 h at 110 °C. Following drying under nitrogen, the hydrolysate was mixed thoroughly with methanol to completely remove the excess trifluoroacetic acid. Subsequently, the hydrolysate was diluted with ultrapure water, and the injection volume was 1 mL. The mobile phase consisted of two solvents, in which mobile phase A was sodium hydroxide and mobile phase B was sodium acetate in sodium hydroxide. The flow rate was set at 0.4 mL/min and the column temperature was set at 30 °C. The monosaccharide composition was determined by matching them with the retention times of standard monosaccharides. Accordingly, the monosaccharides in the PCP-100 were quantified based on the peak areas of the standard monosaccharides, including fucose, rhamnose, arabinose, galactose, glucose, xylose, mannose, ribose, galacturonic acid, and glucuronic acid.

#### 2.6.5. Crystallinity Analysis

The crystalline structure of the PCP-100 was measured using X-ray diffraction (XRD) (Smartlab-3kw, Rigaku, Tokyo, Japan). After being fully ground, the PCP-100 was fixed on an XRD plate and scanned from 6° to 90° at a rate of 2°/min using copper as the target. 

#### 2.6.6. Thermal Analysis

The thermal properties of the PCP-100 were measured using a thermogravimetric analyzer (TGA) (Q50, TA, USA). We placed 5 mg of the PCP-100 in the crucible and examined it in the range of 30~600 °C with increments of 10 °C/min and a nitrogen flow rate of 20 mL/min. Otherwise, a differential scanning calorimeter (DSC) (Netzsch, Selb, Germany) was selected to assess the thermal stability. The PCP-100 was scanned from 25 °C to 200 °C at a rate of 10 °C/min with 20 mL/min of nitrogen flow.

#### 2.6.7. Morphological Analysis

The morphological characteristics of the PCP-100 were detected using scanning electron microscopy (SEM) (SU1510, Hitachi, Tokyo, Japan). Before observation, the PCP-100 was first uniformly sprayed onto conductive carbon adhesive tape, and then we performed a gold-spray treatment.

### 2.7. Antioxidant Activities of the PCP-100

#### 2.7.1. ABTS Radical Scavenging Activity

The free-radical scavenging capacity of the PCP-100 was analyzed using an ABTS test with a minor modification [20]. We mixed 7 mM ABTS with potassium persulfate (2.45 mM) and reacted it at 25 °C for 12 h in dark. Then, this reaction solution was diluted until its absorbance reached 0.7 ± 0.05 at 734 nm. Subsequently, different concentrations (0.05~6.4 mg/mL) of Vc or the PCP-100 solutions were prepared as samples, and 10 μL of the samples were incubated with 200 μL of diluent at 25 °C for 6 min. Finally, the absorbance was measured, and the scavenging rate was calculated using following equation:

Scavenging rate (%) = [A_0_ − (A_1_ − A_2_)]/A_0_ × 100, (1)

where A_0_ was the blank absorbance of the sample that was replaced by water, A_1_ was the sample’s absorbance, and A_2_ was the control absorbance of the ABTS that was replaced by water.

#### 2.7.2. DPPH Radical Scavenging Activity

The activity of the PCP-100 on scavenging DPPH radicals was assayed using a previously described method, with some modifications [21]. Different concentrations of Vc or the PCP-100 solutions (0.05~6.4 mg/mL) were prepared as samples. Then, 1.6 mL of 0.2 mM DPPH ethanol solution were fully mixed with 0.4 mL of the samples, and the reaction mixture were incubated in the dark for 30 min. Finally, the absorbance was read at 517 nm, and the scavenging rate was calculated using the following equation:

Scavenging rate (%) = [A_0_ − (A_1_ − A_2_)]/A_0_ × 100, (2)

where A_0_ was the blank absorbance of the sample that was replaced by water, A_1_ was the sample’s absorbance, and A_2_ was the control absorbance of the DPPH that was replaced by ethanol.

#### 2.7.3. Hydroxyl Radical Scavenging Assay

A hydroxyl assay was also performed to assess the free-radical scavenging capacity of the PCP-100 [22]. Briefly, the mixed systems were composed of salicylic acid–ethanol (6 mM, 0.2 mL), various amounts of Vc or PCP-100 (0.05~6.4 mg/mL, 0.2 mL), FeSO_4_ (6 mM, 0.2 mL), and hydrogen peroxide (0.1%, 0.2 mL). After being kept at 37 °C, the absorbance was read at 510 nm, and the scavenging rate was calculated using the following formula:

Scavenging rate (%) = [A_0_ − (A_1_ − A_2_)]/A_0_ × 100, (3)

where A_0_ was the blank absorbance of the sample that was replaced by water, A_1_ was the sample’s absorbance, and A_2_ was the control absorbance of the hydrogen peroxide that was replaced by water.

#### 2.7.4. Reducing Power Assay

To measure the reducing power of the PCP-100, 0.5 mL of potassium ferricyanide trichloroacetic acid (1%, *w*/*v*) and 0.5 mL of phosphate buffer (0.2 M, pH 6.6) were added to 0.2 mL of the various sample concentrations. The reactions were carried out at 50 °C for 20 min and terminated by the addition of trichloroacetic acid (10%, 0.5 mL). Then, 100 μL of the supernatants were collected by centrifugation and mixed thoroughly with water (100 μL) and FeCl_3_ (0.1%, 20 μL). A higher absorbance, which was recorded at the 700 nm, indicated a better reducing power.

### 2.8. Statistical Analysis

The statistical analyses were conducted with Origin, Design-Expert 10, GraphPad Prism 6.0, and SPSS 19.0 software. All data were evaluated by one-way analysis of variance, followed by a Duncan’s test. A *p*-value of < 0.05 was regarded as statistically significant.

## 3. Results and Discussion

### 3.1. Single-Factor Experiment Analysis

**Figure 1:**
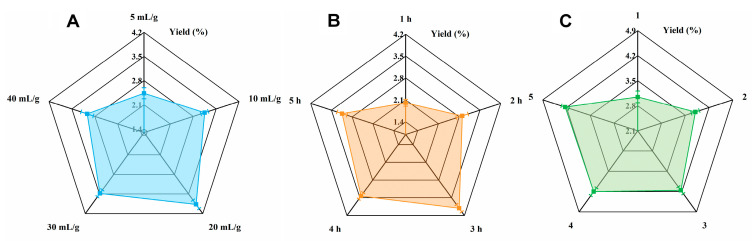
Effects of different factors on the yields of HPCP. (**A**) Liquid–solid ratios. (**B**) Extraction times. (**C**) Number of extraction times.

Different liquid–solid ratios (5, 10, 20, 30, and 40 mL/g) were used during the extraction process for the HPCP. As indicated in Figure 1A, the yields of HPCP increased along with the increases in the liquid–solid ratio from 5 to 20 mL/g. This could be attributed to the fact that at lower liquid–solid ratios, the diffusivity of the polysaccharide particles was enhanced with the increasing solvent volumes [23]. However, further increases in the liquid–solid ratio caused declines in the HPCP yields due to the loss of polysaccharide molecules caused by the presence of large amounts of extraction solvent [24]. Therefore, 20 mL/g was chosen as the central value for the RSM experiments.

The effects of the different extraction times (1, 2, 3, 4, and 5 h) on the yields of HPCP are presented in Figure 1B. It was observed that the yields of HPCP initially increased with increasing the extraction time from 1 to 3 h, but beyond 3 h, the yields declined. In alignment with the results above, the yields of *Platycodon grandiflorus* polysaccharides started to decline after reaching the optimum extraction time [25]. This decline could be attributed to the degradation of the polysaccharides induced by prolonged treatment in a high-temperature environment [7]. Hence, 3 h was selected for further optimization. As shown in Figure 1C, a significant increase in the yield of HPCP occurred when the number of extraction times was increased from one to three. Thereafter, the yields of HPCP increased slowly with the increasing number of extraction times. Based on the yield and cost, three times was regarded as the optimum number of extraction times for further analysis.

### 3.2. Optimization of the Extraction Process of the Polysaccharides

#### 3.2.1. Model Fitting

Based on the above results, liquid–solid ratios ranging from 10 to 30 mL/g, extraction times ranging from 2 to 4 h, and number of extraction times ranging from two to four were selected to optimize the extraction of the HPCP via response surface methodology. Using Design-Expert 10 software to perform the multiple regression analysis, the response and test variables were related by the following quadratic polynomial function:

Y = 4.12 + 0.15X_1_ + 0.22X_2_ + 0.10X_3_ − 0.023X_1_X_2_ − 0.01X_1_X_3_ + 0.05X_2_X_3_ − 0.51X_1_^2^ − 0.60X_2_^2^ − 0.18X_3_^2^. (4)

To evaluate the significance levels of the model equations, *p*-values and F-tests were used. As shown in Table 2, a high F-value (58.97) and a low *p*-value (<0.0001) indicated that the regression model was extremely significant. Additionally, the lack of fit values for the *p*-value and F-value were 0.9260 and 0.15, respectively, which implied that the lack of fit was not significant relative to the pure error. The important value of the adequate precision (23.60) and the minor value of the coefficient variation (C.V.) (2.38%) implied that the experimental values had great reliability [26]. Moreover, the determination coefficient (R^2^) (0.9870) and adjusted determination coefficient (R^2^adj) (0.9702) were close to one, indicating a significant correlation between the predicted and experimental values [7].

#### 3.2.2. Statistical Analysis

**Figure 2:**
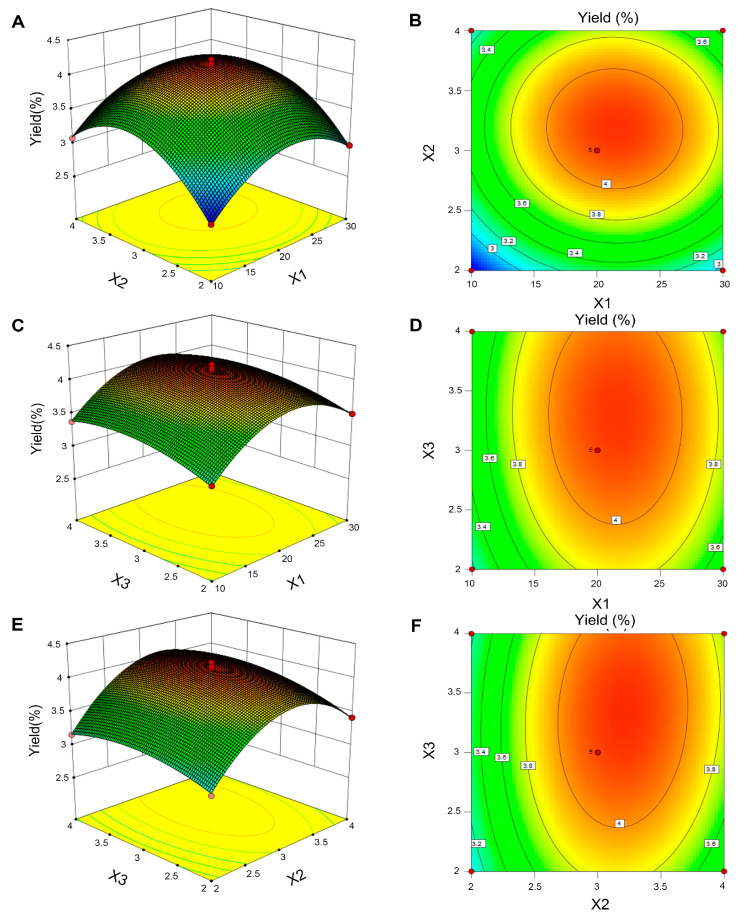
Three-dimensional response surface diagrams and two-dimensional contour diagrams. (**A**,**B**) Liquid–solid ratios (X_1_) and extraction times (X_2_). (**C**,**D**) Liquid**–**solid ratios (X_1_) and number of extraction times (X_3_). (**E**,**F**) Extraction times (X_2_) and number of extraction times (X_3_).

Table 2 demonstrates that the coefficients (X_1_, X_2_, X_3_, X_1_^2^, X_2_^2^, and X_3_^2^) had low *p*-values (*p* < 0.05) and were significant. Furthermore, by examining the order of the *p*-values of the three independent variables, it was clear that extraction time was the most crucial factor affecting the yield of HPCP. The ANOVA results coincided with the analysis of the response surface. Similar to previous study, the response surface and contour plots are presented in Figure 2, which further explicate the correlation between the dependent and independent variables [27].

#### 3.2.3. Verification of the Predictive Model

Based on the aforementioned experimental results, the optimal conditions for extracting polysaccharides from *Penthorum chinense* Pursh were a liquid–solid ratio of 21.37 mL/g, an extraction time of 3.19 h, and 3.32 extraction times, leading to a predicted yield of 4.17%. Considering the convenience of the actual operational procedure, the experimental conditions were modified as follows: liquid–solid ratio of 20 mL/g, extraction time of 3 h, and three extraction times. Subsequently, validation experiments were implemented under the modified conditions, and the actual experimental yield was 4.05 ± 0.12%, which was close to the predicted value. This suggested that the prediction model was appropriate for polysaccharide extraction from *Penthorum chinense* Pursh.

### 3.3. Rheological Properties Analysis 

#### 3.3.1. Effect of Concentration on Rheological Properties

**Figure 3:**
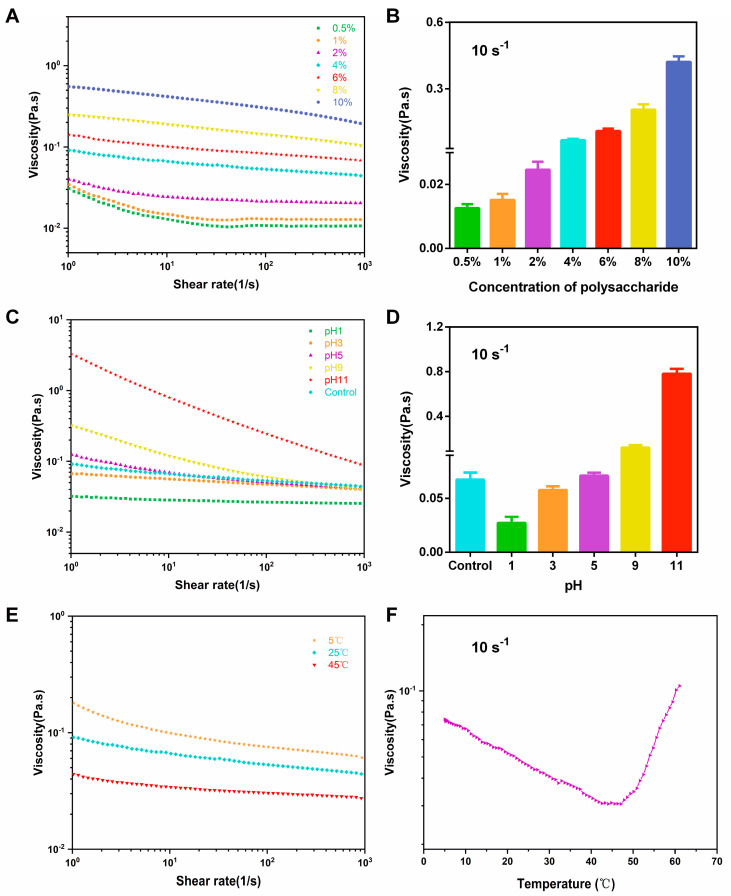
The influence of varying concentrations (**A**,**B**), pH values (**C**,**D**), and temperatures (**E**,**F**) on the rheological properties of the HPCP.

Figure 3A,B depicts the steady rheological properties of the HPCP at different concentrations and shear rates, which ranged from 1 to 1000 s^−1^. The apparent viscosity of the polysaccharides exhibited a concentration-dependent effect, which was related to biopolymer chain aggregation, intermolecular entanglement, and interfacial film formation [28]. At low concentrations (0.5%, 1%, and 2%), the viscosity of the HPCP remained nearly constant as the shear rate increased from 100 to 1000 s^−1^, indicating that the *P. chinense* polysaccharides followed Newtonian flow behavior. However, with higher concentrations (above 2%), the system’s viscosity decreased progressively with increasing shear rates, indicating that the HPCP had shear thinning behavior at these concentrations. In general, higher shear rates can result in a polymer solution’s greater directional flow, with hydrodynamic size changes, leading to a decrease in the apparent viscosity of the polysaccharides [29]. Similar shear-thinning flow behavior has also been observed in other plant-derived polysaccharides, such as those extracted from Dendrobium officinale [30] and cactus pear fruits [31], where the viscosity generally increased with increasing polysaccharide concentrations. The viscosity characteristics of *P. chinense* polysaccharides can offer more convenience in food production and transportation processes.

#### 3.3.2. Effect of pH on Rheological Properties

pH is a key viscosity parameter of the rheological properties of polysaccharide solutions. The rheological properties of *P. chinense* polysaccharides were evaluated at different pH conditions by adjusting 4% HPCP with HCl and NaOH. As shown in Figure 3C,D, both alkaline and acidic conditions differentially altered the rheological behavior of the HPCP such that the viscosity significantly increased when the pH was increased to 11. As an acidic polysaccharide, the 4% HPCP had a pH of 5.69. With a decrease in pH from 5.69 to 1, there was a viscosity change in HPCP that first increased and then decreased, which was consistent with an early study on litchi pulp polysaccharides [32]. This phenomenon may have been due to the addition of small amounts of acid, which strengthened the intermolecular hydrogen bonds, thus increasing the interchain interactions and apparent viscosity [33]. However, strongly acidic conditions may cause the breakdown of polysaccharides or changes in their molecular structure, which can result in a decrease in apparent viscosity [34].

#### 3.3.3. Effect of Temperature on Rheological Properties

It was previously shown that increased temperatures would lead to reductions in the rheological properties, particularly for apparent viscosity [35]. The relationship between apparent viscosity and shear rate at different temperatures is displayed in Figure 3E. The HPCP exhibited the non-Newtonian behavior of shear-thinning at 5 °C, 25 °C, and 45 °C. Meanwhile, higher apparent viscosity was observed at 5 °C, and the HPCP exhibited lower levels of viscosity at 45 °C. The effect of continuously increasing temperatures on the rheological properties at a shear rate of 10 s^−1^ are presented in Figure 3F. Continuous increases in temperature in the range of 5° C to 47 °C led to decreases in the viscosity of the HPCP, but the viscosity showed an increasing tendency when the temperature increased from 47 °C to 60 °C. Given the above results, it could be speculated that *P. chinense* polysaccharides could maintain their structure at low temperatures because of the hydrogen bonding and entanglement between polymers. As the temperature increased, the molecular thermal motion was enhanced, and bonding forces, including hydrophobic, hydrogen, and electrostatic bonds, were inhibited, resulting in a gradual decrease in the apparent viscosity of the system [36]. Meanwhile, the evaporative water loss at high temperatures may have caused an increase in the concentration of the system, which may led to an increase in the apparent viscosity.

#### 3.3.4. Effect of Salt on the Rheological Properties

**Figure 4:**
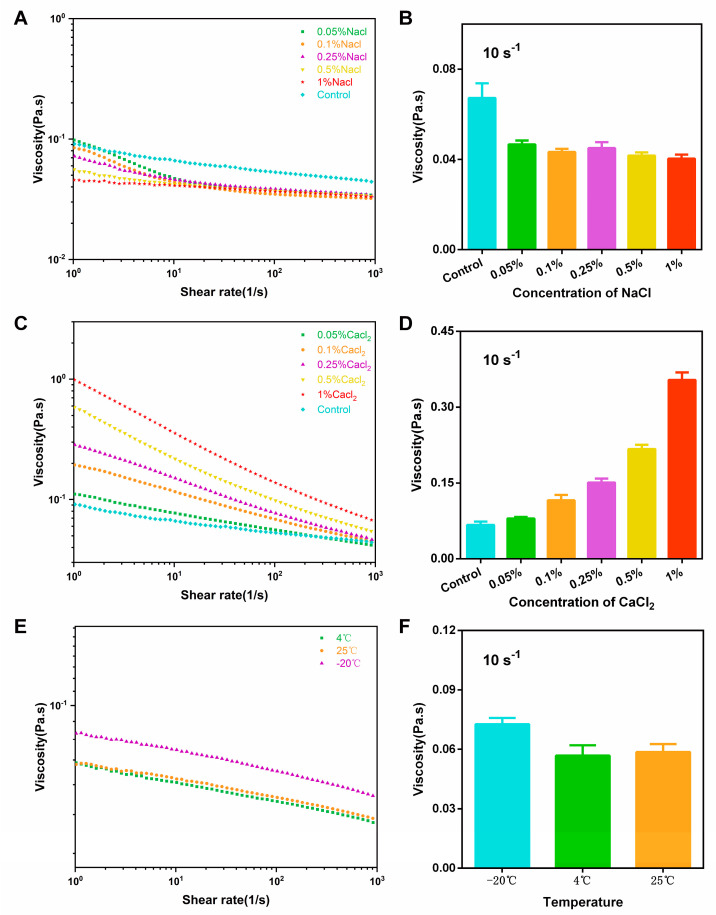
The effects of the addition of NaCl (**A**,**B**) and CaCl_2_ (**C**,**D**) and the freeze–thaw treatments (**E**,**F**) on the rheological properties of the 4% HPCP.

The role of salt in the polysaccharides’ rheological properties is diverse, and it is closely related to the polysaccharides’ structures, as well as concentration and type of salt. The effects of 0.05~1% NaCl and CaCl_2_ on the rheological properties of the 4% HPCP are presented in Figure 4A–D, respectively. The apparent viscosity of the HPCP decreased slightly as the various concentrations of NaCl were added, which could be attributed to the presence of Na^+^ inducing a salting-out effect, thus reducing the polysaccharide concentrations [37]. The non-significant decreasing trend also implied that the *P. chinense* polysaccharides could remain relatively stable in an environment containing NaCl. In this study, CaCl_2_ caused a significant concentration-dependent increase in the polysaccharides’ apparent viscosity. Upon the addition of 1% CaCl_2_, the apparent viscosity of the HPCP increased to 0.35 Pa.s at a 10 s^−1^ shear rate, which was approximately five times higher than that of the initial polysaccharides (0.07 Pa.s). This significant effect of CaCl_2_ on the HPCP might be ascribed to the formation of a network structure by combining divalent ions with carboxyl groups [38]. Beyond this, the addition of CaCl_2_ may also have blocked the electrostatic repulsion between the groups and promoted intermolecular associations, ultimately leading to an increase in the apparent viscosity of the *P. chinense* polysaccharides [39].

#### 3.3.5. Effects of the Freeze–Thaw Treatments on the Rheological Properties

Freeze–thaw variations are widely recognized as unavoidable phenomena in the transport and storage of food, and it will cause food dehydration. The rheological properties of the 4% HPCP, stored at 25 °C, 4 °C, and −20 °C for 24 h, followed by warming at 30 °C for 2 h, were examined to determine the effects of freeze–thaw variations [40]. The results are illustrated in Figure 4E,F. In comparison, there was no obvious difference in the apparent viscosity of the HPCP between the 25 °C and 4 °C treatments. However, a significantly higher apparent viscosity was observed after the −20 °C treatment, implying that the entangled macromolecules during freezing did not fully relax after thawing, ultimately leading to an increase in the apparent viscosity [41]. 

### 3.4. Physicochemical Properties Analysis of the PCP-100 

#### 3.4.1. Chemical Composition Analysis

**Figure 5:**
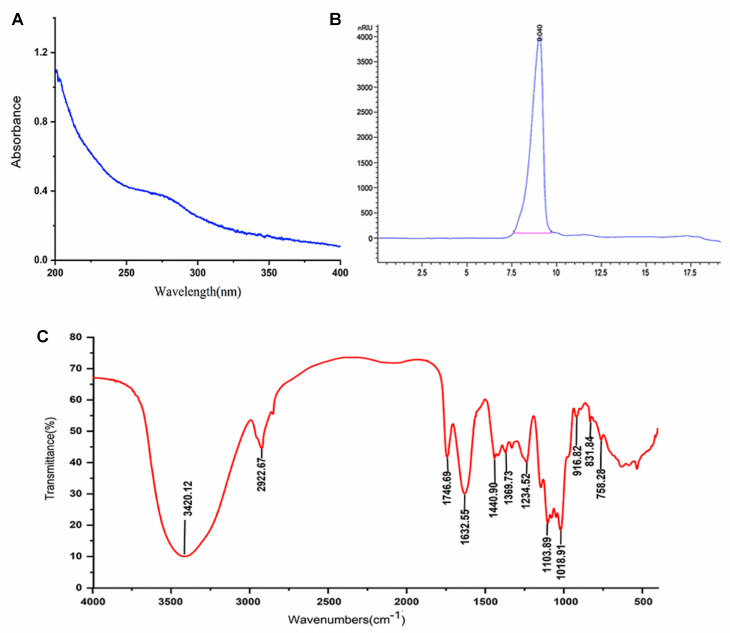
The characteristic analysis of the PCP-100. (**A**) UV spectroscopy. (**B**) HPGPC chromatography. (**C**) FT-IR spectrum.

The chemical composition of the PCP-100 is summarized in Table 3. The total sugar and protein contents of the PCP-100 were 89.01 ± 1.39% and 3.08 ± 0.35%, respectively, as measured by the phenol-sulfuric acid and Bradford methods. In addition, the total phenolic content of the PCP-100 was detected as 0.23 ± 0.01 mg GAE/100 mg. In agreement with the results of the component analysis, the weak absorption peaks at 260~280 nm on the UV spectrum (Figure 5A) also indicated that the PCP-100 contained trace amounts of nucleic acids, proteins, and phenols. Based on the m-hydroxydiphenyl colorimetric assay, the PCP-100 was found to contain uronic acid (29.69 ± 1.15%), indicating that the PCP-100 was an acidic polysaccharide. 

#### 3.4.2. Molecular Weight Analysis

Since molecular weight affects polysaccharide configuration and morphology, its determination is crucial in the study of polysaccharides. As shown in Figure 5B, the PCP-100 was eluted into a single symmetrical peak, indicating that the PCP-100 was a homogeneous polysaccharide. According to the calibration curve of the dextran standards (y = −0.3392x + 9.231, R^2^ = 0.9980), the average molecular weight of the PCP-100 was calculated to be approximately 1.46 × 10^6^ Da, which was lower than that obtained previously [16]. A similar result was previously reported, where *Astragalus membranaceus* polysaccharides extracted at 4 °C had higher molecular weights than those extracted at 100 °C [42]. This may have been attributed to the structural degradation or conformational changes in the polysaccharides induced by high temperatures, which eventually led to decreased molecular weights [43]. Similarly, the average molecular weight of the PCP-100 was lower than that of the polysaccharides prepared at 85 °C [14]. In addition, the average molecular weight of the PCP-100 was higher than that of the polysaccharides reported by Lin et al., which may have been due to differences in the raw material sources and extraction methods [15]. 

#### 3.4.3. FT-IR Spectra Analysis

FT-IR spectroscopy is regarded as an extensively applied technique that reflects information about characteristic groups of polysaccharides. The FT-IR results displayed in Figure 5C reveal the typical absorption peaks of the PCP-100. The two absorption peaks at 3420.12 cm^−1^ and 2922.67 cm^−1^ were assigned to the stretching vibration of O–H and C–H, whereas the peak near 1234.52 cm^−1^ represented the C–H deforming vibration [44]. A peak at 1632.55 cm^−1^ represented the stretching vibration of C=O, and the absorption peaks in the 1000~1200 cm^−1^ range could be ascribed to C–O–C stretching vibrations, indicating that the PCP-100 contained pyranose [45]. These are all common characteristic peaks of polysaccharides. Additionally, the peak detected at 1746.69 cm^−1^ was attributed to the uronic acid component, which was in line with the composition analysis results [46]. The presence of obvious absorption peaks corresponding to α- and β-configurations were observed at 831.84 cm^−1^ and 916.82 cm^−1^, respectively. 

#### 3.4.4. Monosaccharide Composition Analysis

**Figure 6:**
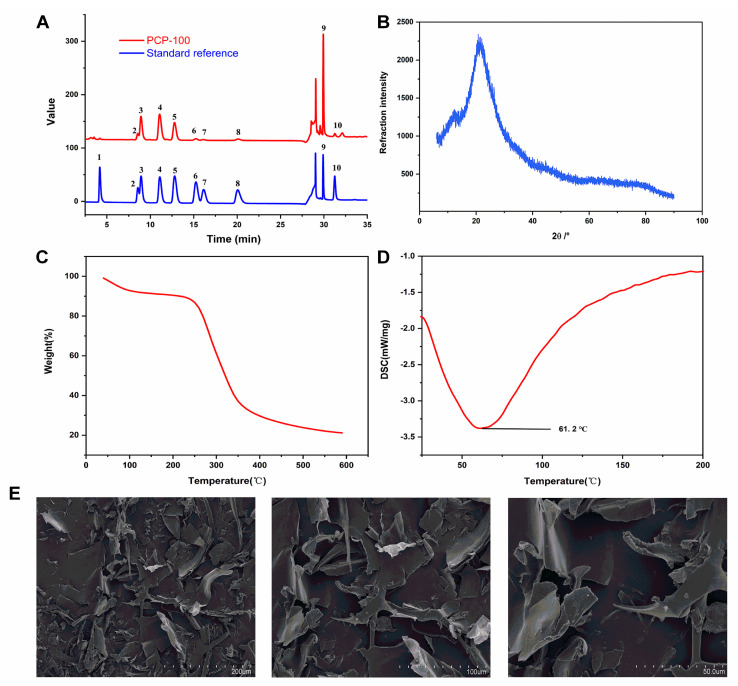
The physicochemical properties analysis of the PCP-100. (**A**) Monosaccharide compositions (1-Fucose, 2-Rhamnose, 3-Arabinose, 4-Galactose, 5-Glucose, 6-Xylose, 7-Mannose, 8-Ribose, 9-Galacturonic acid, and 10-Glucuronic acid). (**B**) XRD pattern. (**C**) TGA curve. (**D**) DSC scan. (**E**) SEM observations.

As showcased in Figure 6A and Table 3, galactose (26.72%) was the predominant monosaccharide in PCP-100, while arabinose (22.87%), glucose (18.99%), and galacturonic acid (21.89%) were also present in relatively high amounts. Otherwise, smaller amounts of rhamnose (4.22%), xylose (1.44%), glucuronic acid (1.10%), mannose (0.67%), and ribose (2.08%) were also detected in the PCP-100. The molar ratios of the above monosaccharides were calculated as 6.32:5.41:4.49:5.18:1:0.34:0.26:0.16:0.49. Despite great similarities in the monosaccharides’ compositions, the PCP-100 showed a striking difference in the monosaccharides’ percentages compared to the *P. chinense* polysaccharides extracted in previous studies. Among them, the PCP-100 had a higher glucose fraction compared to the *P. chinense* polysaccharides extracted at 4 °C [16]. The percentages of xylose and mannose in the PCP-100 were significantly lower than those of the *P. chinense* polysaccharides prepared by enzymatic extraction [15]. In general, the differences in monosaccharide compositions and ratios may have been affected by the origins of the raw materials and the extraction methods used in the different studies.

#### 3.4.5. Crystallinity Analysis

The semi-crystalline and crystalline structures of polysaccharides are strongly related to a variety of biophysical properties, including tensile strength, swelling, and solubility [47]. XRD patterns can deliver information about the bulk phase structures of biomaterials, and thus, they are broadly required in biopolymers analyses. As showcased in Figure 6B, the PCP-100 exhibited a “bun-shaped” peak within the range of 10~30° (2θ), indicating that the PCP-100 had a semi-crystalline structure. Similarly, the polysaccharides derived from *Zingiber officinale Roscoe* [48] and mung bean skin [49] also exhibited semi-crystalline structures.

#### 3.4.6. Thermal Properties Analysis

Heat treatment is an indispensable part of food processing, and so it is important to assess the thermal stability of polysaccharides for their practical applications. The TGA and DSC curves of the PCP-100 are shown in Figure 6C,D. The weightlessness of the PCP-100 in the TGA curve can be attributed the loss of water at approximately 90 °C and the strong thermal decomposition reaction of the polysaccharides in the range of 220~350 °C, respectively. This result was in accordance with those of other studies on polysaccharides [4,50]. In addition, the endothermic peaks for the PCP-100 were captured in a DSC scan, which was ascribed to the evaporation of water [51]. After a comprehensive analysis that combined the TGA and DSC results, the PCP-100 had good thermal stability at temperatures below 200 °C, indicating that it could adapt to the high temperature treatments.

#### 3.4.7. Morphological Properties Analysis

SEM images of the PCP-100 at different magnifications are presented in Figure 6E. The PCP-100 had a pure flaky morphology with an irregular curl, which is believed to be the most common surface morphology of polysaccharides. It has been shown that strong attractions between functional groups may cause the aggregation of polysaccharide chains, leading to the appearance of the flake structure [52]. The smooth surface of the PCP-100 could be captured at a high magnification. Previous studies have shown that polysaccharides’ morphologies are correlated strongly with their rehydration performance, and smooth surfaces could reduce the solubility of polysaccharides [53].

### 3.5. Antioxidant Activities of the PCP-100

There is a growing body of evidence suggesting that polysaccharides derived from natural sources possess potent antioxidant activities both in vitro and in vivo. The antioxidant properties of these polysaccharides are inextricably linked to their reducing abilities, metal ion chelating, and free-radical scavenging potential. Consequently, ABTS, DPPH, and hydroxyl radical scavenging and ferric reducing assays were conducted to estimate the antioxidant activity of the PCP-100. 

#### 3.5.1. ABTS Radical Scavenging Activity

**Figure 7:**
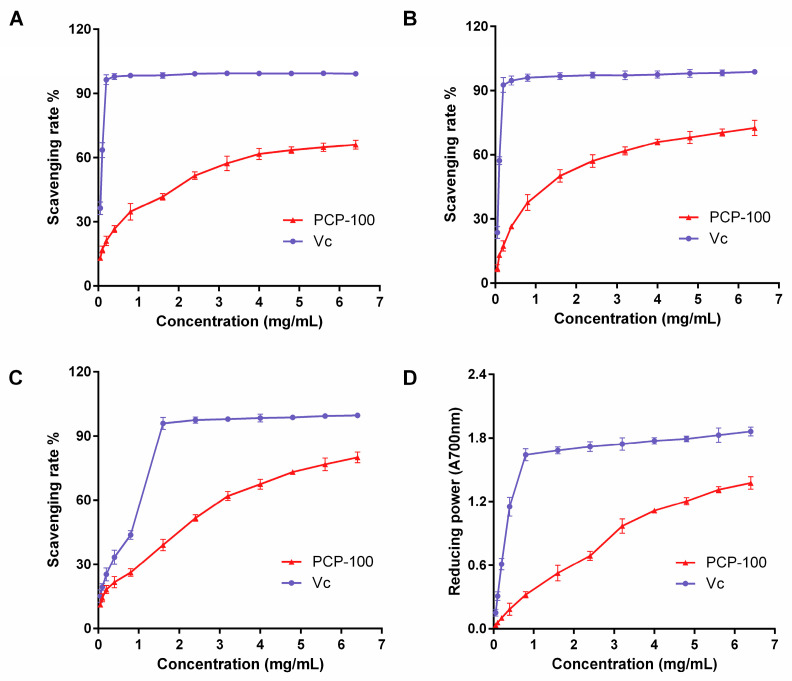
The antioxidant activities of the PCP-100 and ascorbic acid (Vc). (**A**) ABTS radical scavenging activity. (**B**) DPPH radical scavenging activity. (**C**) Hydroxyl radical scavenging activity. (**D**) Reducing power.

Figure 7A illustrates the effect of the PCP-100 on the ABTS radical scavenging ability. A scavenging percentage of 61.68% was observed at a concentration of 4 mg/mL PCP-100, with no significant change in the radical scavenging rate as the concentration was further increased. The half-maximal effective concentration (EC_50_) value of the PCP-100 for ABTS radical scavenging activity was 2.81 mg/mL, which was lower than those of persimmon peel and turmeric polysaccharides [54,55]. In addition, the EC_50_ value of Vc towards the ABTS radical scavenging ability was 0.10 mg/mL. At the same concentration, the scavenging activity of the PCP-100 on ABTS radicals was lower than that of the Vc. Our results aligned with those reported by Chou et al., where *Pholiota nameko* polysaccharides exhibited efficient ABTS radical scavenging in a concentration-dependent manner [56].

#### 3.5.2. DPPH Radical Scavenging Activity

Given its stability, the DPPH radical serves as a useful tool for assessing the free radical scavenging abilities of natural polysaccharides. Figure 7B shows the gradual increase in the DPPH radical scavenging rate from 6.98% to 72.60% with increasing concentrations of the PCP-100 (in the range of 0.05 mg/mL to 6.4 mg/mL). With regard to DPPH radicals, the EC_50_ values of the PCP-100 and Vc were calculated as 1.47 mg/mL and 0.10 mg/mL, respectively. Although the EC_50_ value of the PCP-100 was higher than that of Vc, it was lower than those of the polysaccharides from *pinus koraiensis* and *flammulina velutipes* [57,58]. Yang et al. found that most polysaccharides could scavenge DPPH free radicals due to the hydrogen donation by their hydroxyl groups [59]. The scavenging activity of the PCP-100 against DPPH ions also corroborated this previous finding.

#### 3.5.3. Hydroxyl Radical Scavenging Activity

The hydroxyl radical, one of the most reactive radicals generated during metabolic processes in living organisms, can damage vital biological molecules such as proteins and nucleic acids, disrupt biological equilibrium, and lead to various diseases [60]. Figure 7C illustrates that the concentration of 6.4 mg/mL PCP-100 scavenged 79.96% of the hydroxyl radicals, while Vc scavenged 99.57% at the same concentration. In addition, the EC_50_ value of the PCP-100 for its hydroxyl radical scavenging ability was 3.44 mg/mL, which was lower than that of NLCEPs-1, as obtained from *notarchus leachii freeri* eggs [22].

#### 3.5.4. Reducing Power

It is well known that reducing power is an important indicator for evaluating antioxidant capacity, and so the potassium ferricyanide reduction method is widely used to evaluate antioxidant capacity [61]. As depicted in Figure 7D, the reducing power exhibited a marked increase with increasing concentrations of the PCP-100. However, the reducing ability of V_C_ was significantly better than that of the PCP-100, regardless of the concentration tested.

Previous structure-–activity relationship studies have suggested that the antioxidant activities of polysaccharides may be strongly associated with combinations of several factors, such as functional groups and monosaccharide compositions and contents [62]. Zhang et al. reported that the presence of rhamnose, xylose, and arabinose could enhance the antioxidant activity of polysaccharides [58]. A significant correlation between higher glucose contents and stronger free radical scavenging activities was also confirmed [63]. The antioxidant activities of polysaccharides were confirmed to be affected by the uronic acid contents and especially by the galacturonic acid contents [21]. In addition, natural plant polysaccharides are often combined or mixed with other components such as phenolics, which may be partially responsible for antioxidant activities [64]. Therefore, the strong antioxidant activity exhibited by the PCP-100 may be attributed to its unique monosaccharide composition and proportion. The presence of trace phenolic components in *P. chinense* polysaccharides also promote its antioxidant activity.

## 4. Conclusions

In the present study, water-soluble polysaccharides were obtained from *P. chinense*. On the basis of single-factor and response surface designs, the optimal extraction process of the *P. chinense* polysaccharides were determined to be an extraction time of 3 h, a liquid–solid ratio of 20 mL/g, and three extraction times, at which the yield of polysaccharide was approximately 4.05 ± 0.12% (n = 3). The rheological experiments indicated that the *P. chinense* polysaccharides had the non-Newtonian behavior of shear-thinning. The apparent viscosity of the polysaccharides increased dramatically with increases in concentration and pH and the addition of CaCl_2_, and temperature also significantly influenced the rheological properties of the polysaccharides. After purification by column chromatography, the PCP-100 was mainly composed of glucose (18.99%), arabinose (22.87%), galactose (26.72%), and galacturonic acid (21.89%), with an average molecular weight of 1.46 × 10^6^ Da. The results of the TGA, DSC, SEM, and XRD showed that the PCP-100 possessed a semi-crystalline structure and presented an irregular, sheet-like morphology, and further, that it exhibited excellent thermal stability. Additionally, the antioxidant ability of PCP-100 was confirmed based on its strong reducing power in vitro and its ability to efficiently scavenge DPPH, ABTS, and hydroxyl radicals. In conclusion, our study adds important information to the extraction of *P. chinense* polysaccharides, which can be developed as an antioxidant reagent or natural viscosity modifier in the cosmetic, pharmaceutical, and food industries. However, further studies that focus on the antioxidant activity in vivo and underlying molecular mechanism of *P. chinense* polysaccharides are needed, as are studies on safe doses for human consumption.

## Figures and Tables

**Table 1:** Box-Behnken experimental design and the results.

Number	Liquid–Solid Ratio (X_1_) (mL/g)	Extraction Time (X_2_) (h)	Number of Extraction Times (X_3_)	Yield (%)
1	(1)30	(−1)2	(0)3	2.97
2	(0)20	(0)3	(0)3	4.17
3	(0)20	(1)4	(1)4	3.75
4	(0)20	(−1)2	(−1)2	3.03
5	(1)30	(0)3	(1)4	3.66
6	(1)30	(0)3	(−1)2	3.50
7	(0)20	(0)3	(0)3	4.23
8	(0)20	(−1)2	(1)4	3.16
9	(0)20	(0)3	(0)3	4.11
10	(−1)10	(−1)2	(0)3	2.63
11	(0)20	(0)3	(0)3	3.95
12	(−1)10	(0)3	(−1)2	3.18
13	(0)20	(0)3	(0)3	4.14
14	(−1)10	(0)3	(1)4	3.38
15	(0)20	(1)4	(−1)2	3.42
16	(1)30	(1)4	(0)3	3.33
17	(−1)10	(1)4	(0)3	3.08

**Table 2:** ANOVA for the quadratic response surface model.

Source	Sum of Squares	Degrees of Freedom	Mean Square	F-Value	*p*-Value	Significance
Model	3.71	9	0.41	58.97	<0.0001	**
X_1_	0.18	1	0.18	25.35	0.0015	**
X_2_	0.4	1	0.40	57.36	0.0001	**
X_3_	0.084	1	0.084	12.04	0.0104	*
X_1_X_2_	0.002	1	0.002	0.29	0.6069	
X_1_X_3_	4 × 10^−4^	1	4 × 10^−4^	0.057	0.8177	
X_2_X_3_	0.01	1	0.01	1.43	0.2704	
X_1_^2^	1.11	1	1.11	159.17	<0.0001	**
X_2_^2^	1.53	1	1.53	219.82	<0.0001	**
X_3_^2^	0.13	1	0.13	18.73	0.0034	**
Residual	0.049	7	0.007	-	-	
Pure error	0.044	4	0.011	-	-	-
Lack of fit	0.0049	3	0.002	0.15	0.9260	
Cor Total	3.75	16	-	-	-	
R^2^	0.9870					
R^2^adj	0.9702					
Adequate precision	23.60					
C.V. (%)	2.38					

Note: * *p* < 0.05 was considered significant and ** *p* < 0.01 was considered extremely significant.

**Table 3:** Physicochemical properties and monosaccharide composition of the PCP-100.

Item	Value
Physicochemical property	
Polysaccharide content (%)	89.01 ± 1.39
Uronic acid content (%)	29.69 ± 1.15
Protein content (%)	3.08 ± 0.35
Total phenol content (mg GAE/ 100 mg)	0.23 ± 0.01
Molecular weight (Da)	1.46 × 10^6^
Monosaccharide composition (%)/molar ratio	
Rhamnose	4.22/1.00
Arabinose	22.87/5.41
Galactose	26.72/6.32
Glucose	18.99/4.49
Xylose	1.44/0.34
Mannose	0.67/0.16
Ribose	2.08/0.49
Galacturonic acid	21.89/5.18
Glucuronic acid	1.10/0.26

## Data Availability

Data is contained within the article.

## References

[1] Wang T., Li Q., Bi K. (2018). Bioactive flavonoids in medicinal plants: Structure, activity and biological fate. Asian J. Pharm. Sci..

[2] Elekofehinti O.O. (2015). Saponins: Anti-diabetic principles from medicinal plants—A review. Pathophysiology.

[3] Kuang S., Liu L., Hu Z., Luo M., Fu X., Lin C., He Q. (2023). A review focusing on the benefits of plant-derived polysaccharides for osteoarthritis. Int. J. Biol. Macromol..

[4] Wang L., Zhao Z., Zhao H., Liu M., Lin C., Li L., Ma B. (2022). Pectin polysaccharide from Flos Magnoliae (Xin Yi, Magnolia biondii Pamp. flower buds): Hot-compressed water extraction, purification and partial structural characterization. Food Hydrocoll..

[5] Yuan Q., Lin S., Fu Y., Nie X.-R., Liu W., Su Y., Han Q.-H., Zhao L., Zhang Q., Lin D.-R. (2019). Effects of extraction methods on the physicochemical characteristics and biological activities of polysaccharides from okra (Abelmoschus esculentus). Int. J. Biol. Macromol..

[6] Jiao X., Li F., Zhao J., Wei Y., Zhang L., Wang H., Yu W., Li Q. (2023). Structural diversity and physicochemical properties of polysaccharides isolated from pumpkin (*Cucurbita moschata*) by different methods. Food Res. Int..

[7] Gu J., Zhang H., Zhang J., Wen C., Zhou J., Yao H., He Y., Ma H., Duan Y. (2020). Optimization, characterization, rheological study and immune activities of polysaccharide from *Sagittaria sagittifolia* L. Carbohydr. Polym..

[8] Capitani M.I., Corzo-Rios L.J., Chel-Guerrero L.A., Betancur-Ancona D.A., Nolasco S.M., Tomás M.C. (2015). Rheological properties of aqueous dispersions of chia (*Salvia hispanica* L.) mucilage. J. Food Eng..

[9] Zhou Y., Chen X., Chen T., Chen X. (2022). A review of the antibacterial activity and mechanisms of plant polysaccharides. Trends Food Sci. Technol..

[10] Sun Z.L., Zhang Y.Z., Zhang F., Zhang J.W., Zheng G.C., Tan L., Wang C.Z., Zhou L.D., Zhang Q.H., Yuan C.S. (2018). Quality assessment of Penthorum chinense Pursh through multicomponent qualification and fingerprint, chemometric, and antihepatocarcinoma analyses. Food Funct..

[11] Wang A., Li M., Huang H., Xiao Z., Shen J., Zhao Y., Yin J., Kaboli P.J., Cao J., Cho C.H. (2020). A review of Penthorum chinense Pursh for hepatoprotection: Traditional use, phytochemistry, pharmacology, toxicology and clinical trials. J. Ethnopharmacol..

[12] Yin J., Ren W., Wei B., Huang H., Li M., Wu X., Wang A., Xiao Z., Shen J., Zhao Y. (2020). Characterization of chemical composition and prebiotic effect of a dietary medicinal plant Penthorum chinense Pursh. Food Chem..

[13] Wang A., Lin L., Wang Y. (2015). Traditional Chinese Herbal Medicine Penthorum chinense Pursh: A Phytochemical and Pharmacological Review. Am. J. Chin. Med..

[14] Xu Z., Wang B., Fu L., Wang H., Liu J., Zhou L., Yuan M., Ding C. (2019). Optimization Extraction, Purification and Antioxidant Activities of Polysaccharides from *Penthorum Chinense* Pursh. J. Food Eng..

[15] Lin L.-M., Zhao L.-J., Deng J., Xiong S.-H., Tang J., Li Y.-M., Xia B.-H., Liao D.-F. (2018). Enzymatic Extraction, Purification, and Characterization of Polysaccharides from Penthorum chinense Pursh: Natural Antioxidant and Anti-Inflammatory. BioMed Res. Int..

[16] Chen Y., Chen P., Liu H., Zhang Y., Zhang X. (2023). Penthorum chinense Pursh polysaccharide induces a mitochondrial-dependent apoptosis of H22 cells and activation of immunoregulation in H22 tumor-bearing mice. Int. J. Biol. Macromol..

[17] Guo Y., Ye Q., Yang S., Wu J., Ye B., Wu Y., Huang Z., Zheng C. (2019). Therapeutic effects of polysaccharides from *Anoectochilus roxburghii* on type II collagen-induced arthritis in rats. Int. J. Biol. Macromol..

[18] Blumenkrantz N., Asboe-Hansen G. (1973). New method for quantitative determination of uronic acids. Anal. Biochem..

[19] Bradford M.M. (1976). A rapid and sensitive method for the quantitation of microgram quantities of protein utilizing the principle of protein-dye binding. Anal. Biochem..

[20] Raza A., Li F., Xu X., Tang J. (2017). Optimization of ultrasonic-assisted extraction of antioxidant polysaccharides from the stem of Trapa quadrispinosa using response surface methodology. Int. J. Biol. Macromol..

[21] Pan F., Su T.-J., Liu Y., Hou K., Chen C., Wu W. (2018). Extraction, purification and antioxidation of a polysaccharide from Fritillaria unibracteata var. wabuensis. Int. J. Biol. Macromol..

[22] Pan Q., Sun Y., Li X., Zeng B., Chen D. (2021). Extraction, structural characterization, and antioxidant and immunomodulatory activities of a polysaccharide from Notarchus leachii freeri eggs. Bioorg. Chem..

[23] Wang K., Guo J., Cheng J., Zhao X., Ma B., Yang X., Shao H. (2021). Ultrasound-assisted extraction of polysaccharide from spent Lentinus edodes substrate: Process optimization, precipitation, structural characterization and antioxidant activity. Int. J. Biol. Macromol..

[24] Zhang H., Xie G., Tian M., Pu Q., Qin M. (2016). Optimization of the Ultrasonic-Assisted Extraction of Bioactive Flavonoids from *Ampelopsis grossedentata* and Subsequent Separation and Purification of Two Flavonoid Aglycones by High-Speed Counter-Current Chromatography. Molecules.

[25] Dong X.-d., Liu Y.-n., Yu S.-s., Ji H.-y., Feng Y.-y., Liu A., Yu J. (2021). Extraction, optimization, and biological activities of a low molecular weight polysaccharide from Platycodon grandiflorus. Ind. Crops Prod..

[26] Mzoughi Z., Abdelhamid A., Rihouey C., Le Cerf D., Bouraoui A., Majdoub H. (2018). Optimized extraction of pectin-like polysaccharide from Suaeda fruticosa leaves: Characterization, antioxidant, anti-inflammatory and analgesic activities. Carbohydr. Polym..

[27] Yu P., Chao X. (2013). Statistics-based optimization of the extraction process of kelp polysaccharide and its activities. Carbohydr. Polym..

[28] Wang Y.-X., Yin J.-Y., Huang X.-J., Nie S.-P. (2020). Structural characteristics and rheological properties of high viscous glucan from fruit body of Dictyophora rubrovolvata. Food Hydrocoll..

[29] Li Y., Wang X., Lv X., Wang X., Wang X., Cui J., Yan M. (2020). Extractions and rheological properties of polysaccharide from okra pulp under mild conditions. Int. J. Biol. Macromol..

[30] Wang B., Zhang W., Bai X., Li C., Xiang D. (2020). Rheological and physicochemical properties of polysaccharides extracted from stems of Dendrobium officinale. Food Hydrocoll..

[31] Morales-Martínez Y., López-Cuellar M.d.R., Chavarría-Hernández N., Rodríguez-Hernández A.I. (2018). Rheological behaviour of acetylated pectins from cactus pear fruits (*Opuntia albicarpa* and *O. matudae*). Food Hydrocoll..

[32] Huang F., Liu Y., Zhang R., Dong L., Yi Y., Deng Y., Wei Z., Wang G., Zhang M. (2018). Chemical and rheological properties of polysaccharides from litchi pulp. Int. J. Biol. Macromol..

[33] Dikeman C.L., Fahey G.C. (2006). Viscosity as Related to Dietary Fiber: A Review. Crit. Rev. Food Sci. Nutr..

[34] Fabek H., Messerschmidt S., Brulport V., Goff H.D. (2014). The effect of in vitro digestive processes on the viscosity of dietary fibres and their influence on glucose diffusion. Food Hydrocoll..

[35] Li K., Zhu L., Li H., Zhu Y., Pan C., Gao X., Liu W. (2019). Structural characterization and rheological properties of a pectin with anti-constipation activity from the roots of *Arctium lappa* L. Carbohydr. Polym..

[36] Shao H., Zhang H., Tian Y., Song Z., Lai P.F.H., Ai L. (2019). Composition and Rheological Properties of Polysaccharide Extracted from Tamarind (*Tamarindus indica* L.) Seed. Molecules.

[37] Yang B., Wu Q., Luo Y., Yang Q., Chen G., Wei X., Kan J. (2019). Japanese grape (*Hovenia dulcis*) polysaccharides: New insight into extraction, characterization, rheological properties, and bioactivities. Int. J. Biol. Macromol..

[38] Mierczyńska J., Cybulska J., Sołowiej B., Zdunek A. (2015). Effect of Ca^2+^, Fe^2+^ and Mg^2+^ on rheological properties of new food matrix made of modified cell wall polysaccharides from apple. Carbohydr. Polym..

[39] Xu G.-Y., Liao A.-M., Huang J.-H., Zhang J.-G., Thakur K., Wei Z.-J. (2019). The rheological properties of differentially extracted polysaccharides from potatoes peels. Int. J. Biol. Macromol..

[40] Lin L., Shen M., Liu S., Tang W., Wang Z., Xie M., Xie J. (2018). An acidic heteropolysaccharide from Mesona chinensis: Rheological properties, gelling behavior and texture characteristics. Int. J. Biol. Macromol..

[41] Liu J., Wang B., Lin L., Zhang J., Liu W., Xie J., Ding Y. (2014). Functional, physicochemical properties and structure of cross-linked oxidized maize starch. Food Hydrocoll..

[42] Yu J., Ji H., Yang Z., Liu A. (2019). Relationship between structural properties and antitumor activity of Astragalus polysaccharides extracted with different temperatures. Int. J. Biol. Macromol..

[43] Zhao J., Liang K., Zhong H., Liu S., He R., Sun P. (2022). A cold-water polysaccharide-protein complex from Grifola frondosa exhibited antiproliferative activity via mitochondrial apoptotic and Fas/FasL pathways in HepG2 cells. Int. J. Biol. Macromol..

[44] Guan Y., Sun H., Chen H., Li P., Shan Y., Li X. (2021). Physicochemical characterization and the hypoglycemia effects of polysaccharide isolated from Passiflora edulis Sims peel. Food Funct..

[45] Nie C., Zhu P., Ma S., Wang M., Hu Y. (2018). Purification, characterization and immunomodulatory activity of polysaccharides from stem lettuce. Carbohydr. Polym..

[46] Wan X., Jin X., Xie M., Liu J., Gontcharov A.A., Wang H., Lv R., Liu D., Wang Q., Li Y. (2020). Characterization of a polysaccharide from Sanghuangporus vaninii and its antitumor regulation via activation of the p53 signaling pathway in breast cancer MCF-7 cells. Int. J. Biol. Macromol..

[47] Ji X., Yan Y., Hou C., Shi M., Liu Y. (2020). Structural characterization of a galacturonic acid-rich polysaccharide from *Ziziphus Jujuba cv. Muzao*. Int. J. Biol. Macromol..

[48] Chen X., Wang Z., Kan J. (2021). Polysaccharides from ginger stems and leaves: Effects of dual and triple frequency ultrasound assisted extraction on structural characteristics and biological activities. Food Biosci..

[49] Chen S., Qin L., Xie L., Yu Q., Chen Y., Chen T., Lu H., Xie J. (2022). Physicochemical characterization, rheological and antioxidant properties of three alkali-extracted polysaccharides from mung bean skin. Food Hydrocoll..

[50] Karimi S., Ghanbarzadeh B., Roufegarinejad L., Falcone P.M. (2021). Polysaccharide extracted from *Althaea officinalis* L. root: New studies of structural, rheological and antioxidant properties. Carbohydr. Res..

[51] Kong L., Yu L., Feng T., Yin X., Liu T., Dong L. (2015). Physicochemical characterization of the polysaccharide from Bletilla striata: Effect of drying method. Carbohydr. Polym..

[52] Li Q., Wang W., Zhu Y., Chen Y., Zhang W., Yu P., Mao G., Zhao T., Feng W., Yang L. (2017). Structural elucidation and antioxidant activity a novel Se-polysaccharide from Se-enriched Grifola frondosa. Carbohydr. Polym..

[53] Xu J., Chen Z., Liu P., Wei Y., Zhang M., Huang X., Peng L., Wei X. (2021). Structural characterization of a pure polysaccharide from Bletilla striata tubers and its protective effect against H2O2-induced injury fibroblast cells. Int. J. Biol. Macromol..

[54] Cui Y., Chen Y., Wang S., Wang S., Yang J., Ismael M., Wang X., Lü X. (2023). Purification, structural characterization and antioxidant activities of two neutral polysaccharides from persimmon peel. Int. J. Biol. Macromol..

[55] Zhu Z., Chen J., Chen Y., Ma Y., Yang Q., Fan Y., Fu C., Limsila B., Li R., Liao W. (2022). Extraction, structural characterization and antioxidant activity of turmeric polysaccharides. LWT.

[56] Chou C.-H., Sung T.-J., Hu Y.-N., Lu H.-Y., Yang L.-C., Cheng K.-C., Lai P.-S., Hsieh C.-W. (2019). Chemical analysis, moisture-preserving, and antioxidant activities of polysaccharides from *Pholiota nameko* by fractional precipitation. Int. J. Biol. Macromol..

[57] Liu Y., Zhang B., Ibrahim S.A., Gao S.-S., Yang H., Huang W. (2016). Purification, characterization and antioxidant activity of polysaccharides from *Flammulina velutipes* residue. Carbohydr. Polym..

[58] Zhang H., Zou P., Zhao H., Qiu J., Regenstein J.M., Yang X. (2021). Isolation, purification, structure and antioxidant activity of polysaccharide from pinecones of *Pinus koraiensis*. Carbohydr. Polym..

[59] Yang B., Zhao M., Prasad K.N., Jiang G., Jiang Y. (2010). Effect of methylation on the structure and radical scavenging activity of polysaccharides from longan (*Dimocarpus longan* Lour.) fruit pericarp. Food Chem..

[60] Sakai T., Imai J., Ito T., Takagaki H., Ui M., Hatta S. (2017). The novel antioxidant TA293 reveals the role of cytoplasmic hydroxyl radicals in oxidative stress-induced senescence and inflammation. Biochem. Biophys. Res. Commun..

[61] Zhang Z., Lv G., Pan H., Shi L., Fan L. (2011). Optimization of the Microwave-Assisted Extraction Process for Polysaccharides in Himematsutake (*Agaricus blazei Murrill*) and Evaluation of Their Antioxidant Activities. Food Sci. Technol. Res..

[62] Jiang Y.-Y., Yu J., Li Y.-B., Wang L., Hu L., Zhang L., Zhou Y.-H. (2019). Extraction and antioxidant activities of polysaccharides from roots of *Arctium lappa* L. Int. J. Biol. Macromol..

[63] Cai L., Chen B., Yi F., Zou S. (2019). Optimization of extraction of polysaccharide from dandelion root by response surface methodology: Structural characterization and antioxidant activity. Int. J. Biol. Macromol..

[64] Yuan L., Qiu Z., Yang Y., Liu C., Zhang R. (2022). Preparation, structural characterization and antioxidant activity of water-soluble polysaccharides and purified fractions from blackened jujube by an activity-oriented approach. Food Chem..

